# Fibrin tissue adhesive reduces postoperative blood loss in total knee arthroplasty

**DOI:** 10.1007/s10195-012-0198-7

**Published:** 2012-05-16

**Authors:** Luigi Sabatini, Andrea Trecci, Daniele Imarisio, Marco Davide Uslenghi, Giuseppe Bianco, Roberto Scagnelli

**Affiliations:** Orthopedics and Traumatology Department, Ospedale Civile di Saluzzo, Via Spielberg 58, 12037 Saluzzo, CN Italy

**Keywords:** Fibrin tissue adhesive, Blood loss, Total knee arthroplasty, Fibrin sealant

## Abstract

**Background:**

Blood transfusion is often required in total knee replacement; various methods of blood preservation have been studied. The best solution is to reduce the loss of blood during and after surgery.

**Materials and methods:**

We designed this study to evaluate the hemostatic efficacy and safety of fibrin tissue adhesive (Quixil) in patients receiving total knee arthroplasty [low contact stress (LCS, DePuy, Warsaw, IN, US) cementless total knee replacement (TKR)] with a prospective, randomized, standard treatment controlled study. Thirty-five patients were randomized to receive treatment with fibrin tissue adhesive (treatment group), and 35 were randomized to be managed with postoperative blood recovery and reinfusion (control group). Blood loss in suction drain, decrease in hemoglobin values, and transfusions were recorded.

**Results:**

A significant reduction in apparent total blood loss was detected in the treatment group compared with the control group. There was also a lower decrease in hemoglobin level, although this difference was not significant. When fibrin tissue adhesive was administered, the need for transfusions was lower. No major adverse events were recorded in our series.

**Conclusions:**

Fibrin tissue adhesive reduced blood loss in TKR and seemed to significantly reduce the need for blood transfusion. Fibrin tissue adhesive can be an appropriate solution to enhance hemostasis and vessel sealing at the operative site in TKR, in order to reduce blood loss after surgery and the risk of complications.

## Introduction

Total knee replacement (TKR) is associated with major intra- and postoperative blood loss (approximately 800–1,400 ml), and blood transfusion is frequently required [1]. Use of tourniquet may reduce intraoperative blood loss, but postoperative blood loss can still be considerable.

Decreasing this postoperative blood loss may reduce patient morbidity [2, 3], length of hospitalization, and costs by eliminating the need for transfusion.

Various methods of blood preservation have been studied, in order to avoid transmission of viral diseases and transfusion reactions; the various perioperative methods include hemodilution, intraoperative and postoperative blood salvage and reinfusion [4], hypotensive or epidural anesthesia, and transfusion of predonated autologous blood [5].

Obviously, the most appropriate solution is to enhance hemostasis and vessel sealing at the operative site. Plasma proteins were used in the past. Modern treatment with fibrin tissue adhesive, also known as fibrin glue or fibrin sealant, consists in application of plasma fibrinogen mixed with thrombin to form an adhesive fibrin clot. Since 1972, fibrin sealants have been increasingly used as hemostatic and sealing agents in a variety of surgical specialties, including, recently, total knee replacement [6].

Because of a lack of solid evidence concerning safety and efficacy, blood-bank products and bovine thrombin concentrates have been used extensively for many years.

Fibrin tissue adhesive is composed of two main components: fibrinogen and thrombin. When mixed together, they mimic the last step of the coagulation cascade: thrombin activates fibrinogen to polymerize to an unstable clot, and factor XIII, which is present in the fibrinogen concentrate and is activated by thrombin (factor XIIIa), stabilizes the clot by catalyzing cross-links between the fibrin molecules. Factor XIIIa also forms cross-links between natural plasmin inhibitors (which copurify with fibrinogen) and the fibrinogen mash to enhance clot resistance against fibrinolysis. Some products contain additional fibrinolytic inhibitors, such as bovine aprotinin or tranexamic acid [7], although the contribution of such additives is controversial [8].

The desire to avoid any bovine products led to the development of a second-generation fibrin glue which replaced bovine aprotinin with tranexamic acid as the antifibrinolytic agent; safety issues concerning tranexamic acid led the manufacturer to develop a modified version without the antifibrinolytic agent [9]. In addition, successful application of tissue adhesive needs experience and training [10].

There are numerous publications concerning good results of use of fibrin tissue adhesive in all fields of surgery, but many reports are uncontrolled studies and some trials show no benefits but harmful effects [11]. In orthopedic surgery, the literature is poor and there are few reports of total knee replacement, for which fibrin glue is not yet used routinely.

## Materials and methods

We designed this study to evaluate the hemostatic efficacy of fibrin tissue adhesive in patients receiving total knee arthroplasty. This is a prospective, randomized, standard treatment controlled study, where the control group had postoperative blood recovery and reinfusion. Both fibrin tissue adhesive and postoperative blood reinfusion were already approved for use in our hospital. We decided to evaluate the differences between the two procedures, and the efficacy of fibrin adhesive [5]. The protocol used conformed to the Declaration of Helsinki, and we notified the Ethical Review Board. All patients signed informed written consent to either procedure before being included in the study. All patients were enrolled in a program of autologous blood recovery; for each patient in good health conditions and with good hemoglobin values, we prepared two units of autologous blood.

We evaluated 70 patients treated for osteoarthritis of the knee with total knee cementless arthroplasty from April 2009 to April 2010. Exclusion criteria were cemented TKR, current infection, any kind of cancer, and coagulation pathology, in order to create two similar groups: treatment and control.

Thirty-five patients were randomized to receive treatment with fibrin tissue adhesive (the treatment group), and 35 were randomized to be managed with postoperative blood recovery and reinfusion (control group, Di-deco).

The mean age in the fibrin tissue adhesive group was 70.7 ± 6.4 years, while in the Di-deco group it was 70.4 ± 6.7 years (*p* = 0.84).

In our series, 54 patients were female and 16 were male (10 males in the treatment group, 6 in the control group).

The mean presurgery hemoglobin value in the treatment group was 13.5 ± 1.5 g/dl, and in the control group it was 13.2 ± 1.3 g/dl (*p* = 0.34).

The mean surgical time was 79 ± 16 min for the treatment group and 78.2 ± 14.3 min for the control group (*p* = 0.84).

The fibrin tissue adhesive used was Quixil (Omrix Biopharmaceuticals, Belgium), formed by two 5-ml components: cryoprecipitated fibrinogen, and a high concentration of human thrombin dissolved in calcium chloride; an antifibrinolytic agent, tranexamic acid, is added to the fibrinogen as a stabilizer. Both components undergo double viral inactivation. The constituents are placed in separate syringe tubes and mixed by connecting them together to a single lumen, through which the glue is expelled as a high-pressure spray.

All total knee arthroplasties were managed by the same surgical team; a LCS total knee prosthesis cementless procedure was used for all operations [LCS rotating platform (RP) or LCS anterior–posterior glide (APG) with posterior cruciate sparing], all performed by medial parapatellar approach in a bloodless field with use of tourniquet until prosthesis insertion (Tables 1, 2).

**Table 1:** Group features

Patient no.	Di-deco			Quixil		
	Sex	Age (years)	Hb before surgery (g/dl)	Sex	Age (years)	Hb before surgery (g/dl)
1	F	67	11.2	F	78	10.5
2	F	69	12	F	78	12.9
3	F	76	13.5	M	69	12.9
4	F	79	11.1	F	78	12.7
5	M	67	12.8	F	63	13.4
6	F	64	13.8	F	53	13.6
7	F	65	12.2	F	73	12.4
8	F	71	11.9	F	75	15.8
9	F	86	15	M	74	15.4
10	F	74	14.7	F	74	15.5
11	F	70	11.7	M	77	13.4
12	F	70	13.9	M	82	14.1
13	F	59	13.1	F	69	12.4
14	F	76	11.7	M	63	15.2
15	F	73	12.3	M	74	15
16	F	79	13.3	F	77	13.3
17	M	68	14.4	m	78	17.5
18	F	71	13.8	F	64	13.1
19	F	73	13.8	F	66	12
20	F	56	11.9	F	75	15.2
21	F	80	13.6	F	78	13.6
22	F	69	11.7	M	77	13.6
23	F	66	11.5	M	60	12.2
24	F	79	14.6	F	67	12.6
25	M	76	13.6	F	71	12
26	M	75	14.4	F	68	11.4
27	M	54	13.2	F	62	14.8
28	F	78	14.2	F	69	13.8
29	F	70	15.8	F	61	14.2
30	F	66	12.4	F	70	13.6
31	F	70	14.4	F	72	10.2
32	F	66	12.9	M	73	14.7
33	M	70	15.6	F	69	13.8
34	F	65	14.6	F	68	14.2
35	F	67	11.7	F	70	12.6
	6 males			10 males		
Mean		70.4	13.2085714		70.7142857	13.5314286
Median		70	13.3		71	13.6
SD		6.78753182	1.29555154		6.4606111	1.51167446

*SD* standard deviation

**Table 2:** Postoperative findings

Patient no.	Di-deco				Quixil			
	Blood loss (ml)	Hb decrease (g/dl)	Transfusions	Surgical time (min)	Blood loss (ml)	Hb decrease (g/dl)	Transfusions	Surgical time (min)
1	840	2.5	2a^a^	90	1,290	0.5	—	90
2	1,100	3.3	1a	70	650	2.8	—	80
3	1,800	3.6	1h^b^	70	1,420	0.9	—	90
4	1,470	4.4	2h	90	620	2.4	—	70
5	2,650	1.6	2h	115	470	2.8	—	90
6	700	2.7	—	100	400	3.5	—	110
7	1,650	−0.2	—	60	500	1.7	—	70
8	1,480	3.7	2a	60	1,090	5.5	1a	60
9	2,070	2.5	—	70	1,090	2.6	—	70
10	1,120	3.9	—	90	1,050	2.9	—	80
11	1,750	1.6	—	100	650	2.7	—	85
12	1,710	3.8	1h	105	1,150	2.7	1h	90
13	760	5.1	3h	90	910	1.8	—	75
14	800	3	1h	95	1,010	3.8	—	70
15	500	2.9	—	70	650	3.6	—	60
16	700	1.5	—	80	850	2.9	—	60
17	600	3.7	—	80	1,060	2.6	—	65
18	1,200	0.8	—	90	700	2.3	—	100
19	2,350	3.2	1a	60	650	2.8	—	80
20	850	3.4	1h	80	1,000	3.7	—	75
21	800	2.7	—	70	1,070	4.2	—	60
22	1,750	2.9	1h	75	1,150	0.9	—	70
23	2,050	3.1	1h	70	1,480	1.7	1a	100
24	1,750	0.9	—	65	570	1.6	—	110
25	1,850	0	—	70	1,150	0	—	80
26	1,750	3.3	—	90	850	1.6	—	65
27	1,000	0	—	60	1,030	4.8	—	120
28	1,050	3.1	—	70	850	2.3	—	80
29	1,250	4.1	—	65	950	3.6	—	70
30	1,000	2.3	—	80	800	3.2	—	70
31	1,800	2	—	70	370	1.4	1a	70
32	1,100	3.7	2a	70	1,020	2.6	—	65
33	2,050	2.5	—	60	400	2.6	—	65
34	1,050	4	—	70	1,200	2.6	—	65
35	1,800	3.8	1a	90	650	1.6	2h	105
Mean	1,375.71429	2.72571429		78.2857143	878.571429	2.54857143		79
Median	1,250	3		70	910	2.6		75
SD	546.170011	1.28757475		14.3968367	292.485725	1.15970874		16.0788498

^a^Autologous

^b^Homologous

Student *t* test (blood loss): *p* = 1.6558 × 10^−5^

Student *t* test (Hb decrease): *p* = 0.54736077

Student *t* test (Hb levels before surgery): *p* = 0.34083659

Fisher test (blood units administered): 15 patients (Di-deco) versus 6 patients (Quixil): *p* = 0.03

Fisher test (at least two blood units administered): 12 patients (Di-deco) versus 2 patients (Quixil): *p* = 0.006

In the treatment group, after the preparation of the femur and the tibia and before insertion of the prosthesis, the operative field was rinsed of any debris and meticulously dried, then the fibrin tissue adhesive (half of 5 ml of each product component, corresponding to half of the kit) was applied to the back of the knee cavity, the posterior recess, the gutters, and the exposed surfaces of the femur and tibia by topical air pressure spraying with use of a double-syringe spray device from a distance of approximately 15 cm. Then, after prosthesis insertion, remaining product was applied over soft tissues, extensor mechanism, and prepatellar bursa, to cover as much surface area as possible with glue film.

After the tourniquet was deflated (2 min after glue application), we performed hemostasis of major vessels using electrocautery. We then placed two drains: one inside and the other outside of the knee joint.

In the control group, after deflating the tourniquet, we performed hemostasis of major vessels and connected the same two drains to a Di-deco blood recovery device without fibrin tissue adhesive application.

Hemoglobin and hematocrit values were determined preoperatively (the day before operation) and on the first and third postoperative days. Preoperative platelet count, prothrombin time, and activated partial thromboplastin time were determined for all patients.

Intraoperative blood loss was similar in the two groups because of the use of tourniquet and the same operative technique.

Blood loss at the end of the operation was recorded by measuring the volume in the suction apparatus without washing solution; apparent postoperative blood loss was recorded by measuring the volume in the suction drain bottles on the evening of the operation and the first, second, and third postoperative days (until drain removal).

In the control group, the Di-deco device recovered the blood loss for the first 6 h after operation; if the blood amount in this time was sufficient to be washed and prepared (400 ml), it was reinfused. At the end of 6 h, the Di-deco device was replaced with a suction drain. All Di-deco reinfusions were recorded.

In all cases (in the treatment group, and in the control group after Di-deco reinfusion), decisions regarding blood transfusion were based on hemoglobin value (measured 4–6 h after operation, and on the first, third, and fifth postoperative days) and clinical conditions, including also cardiovascular history and patient age, always being used for hemoglobin values lower than 8 g/dl [12]. The decision to administer blood was taken by a surgeon blinded to the patient’s group.

All patients received subcutaneous injection of low-molecular-weight heparin every evening of the period of hospitalization, starting the day before surgery.

Any complications, such as wound complications, fever, prolonged drainage from drain site, and adverse events, were recorded.

After the first postoperative day, patients began continuous passive motion; all patients were allowed to get out of bed after the third postoperative day and started physiotherapy the same day.

The end points of this study are evaluation of the efficacy of fibrin tissue adhesive in reducing blood loss, decrease of hemoglobin on the first postoperative day, and the number of transfused units needed by patients. To evaluate these values we used Student’s *t* test and Fisher’s exact test.

## Results

The treatment and control groups were comparable in terms of patient characteristics such as general health conditions, age, presurgery hemoglobin level (*p* = 0.34), and surgery time. As previously stated, intraoperative blood loss was similar for the two groups because of tourniquet use.

The median apparent postoperative blood loss at drain removal (third postoperative day) was 910 ± 292 ml in the fibrin tissue adhesive group compared with 1,250 ± 546 ml in the control group, and the difference was highly statistically significant (*p* = 0.0000165).

The median decrease in hemoglobin concentration on the first postoperative day was 2.6 ± 1.16 g/dl in the fibrin tissue adhesive group and 3 ± 1.28 g/dl in the control group. The difference in this case was not statistically significant (*p* = 0.55).

The blood transfusion requirements in the fibrin tissue adhesive group also were found to be significantly (*p* = 0.03) lower than those in the control group; only 5 patients in the fibrin tissue adhesive group required blood transfusion, and only 1 patient required two units, whereas 15 patients in the control group required blood transfusion, with 5 requiring two units and 1 requiring three units (for more than one transfusion unit, *p* = 0.0057).

### Adverse events

Fever was the most common adverse event associated with surgery, with no difference in frequency between the two group (six patients in the treatment group, six in the control group). Hematoma was recorded in two cases in the fibrin adhesive group and six patients in the control group (one in the control group needing surgical drainage).

We recorded no superficial wound infection or deep vein thrombosis. We recorded no embolism in our series after application of fibrin tissue adhesive by topical spraying with use of the double-syringe spray device.

## Discussion

Many bleeding problems are involved in TKR, not only intraoperatively but also postoperatively. The amount of blood loss after total knee arthroplasty is often underestimated because the apparent rate is considered instead of the calculated blood loss [13].

Intraoperative use of tourniquet (prolonged ischemia in the limb increases fibrinolytic activity) and suction drainage use can increase blood loss.

In many cases it is necessary to transfuse blood units to avoid a large decrease in hemoglobin values [14, 15]. So, it appears very useful to adopt the most appropriate solution to enhance both hemostasis and safety of recovery and reinfusion [16].

Until last year, in our clinical practice we prepared two predonated units of autologous blood for all patients in good health condition (and less than 75 years old), or prepared two units of homologous blood in the other cases in addition to use of the Di-deco device. The procedure regarding preparation of autologous blood units and the Di-deco device has associated costs, even if they reduce the necessity for homologous blood transfusions, thus reducing costs and risk of infection. While autologous predonated blood is not associated with a risk of viral disease transmission, the rates of administrative error and bacterial overgrowth after infusion of autologous blood (the two factors most frequently associated with immediate posttransfusion death) are comparable to those associated with use of homologous blood [17].

Through the use of fibrin sealant we hoped to prevent the need for recovery and reinfusion due to enhanced hemostasis in the operative field; use of Quixil was our choice, so we evaluated in this study the efficacy and safety of fibrin tissue adhesive in knee replacement as an alternative to use of the Di-deco device.

Blood loss in our patients (in both groups) was similar to mean blood loss reported in literature; a significant reduction in apparent total blood loss was found in the group treated with fibrin tissue adhesive compared with the control group, where the Di-deco device was used. It is important to underline that, in the control group, not all patients reached the amount of blood loss needed to trigger reinfusion until 6 h; in our series, 23 patients (66 %) received blood recovery from Di-deco.

The treatment group had a smaller postoperative mean decrease in hemoglobin level than the control group on the first postoperative day, but this difference was not significant.

Use of fibrin tissue adhesive significantly reduced the total number of units of blood transfused postoperatively to almost one-third of the value in the control group (8 versus 22); it reduced also the rate of patients requiring transfusion to almost one-third (6 versus 15), with only 2 patients requiring more than one unit, compared with 6 in the control group.

Since the decision regarding transfusion and the number of blood units transfused are unreliable values, being influenced by patient conditions and medical decisions, we did not consider these parameters as significant.

Prevention of blood loss, including prevention of concomitant compartmental shift in body fluid, is definitely superior to replacement of lost blood. It is much safer for a patient to receive a multidonor viral-inactivated blood product (fibrin tissue adhesive) than homologous blood that cannot be viral inactivated [18].

The results of our study suggest that use of Quixil in total knee arthroplasty reduces postoperative apparent blood loss, also compared with use of the Di-deco device.

The Italian Agency of Drugs (AIFA) had informed that use of a spray device to apply fibrin tissue adhesive can produce massive embolism (two cases, one fatal, having been reported). They recommend use of a spray device with pressure less than 2.0–2.5 bar, to apply Quixil from a minimal distance of 10–15 cm, and to monitor patients during spray application. We were informed of these recommendations after our study, but we did not find any clinical signs of pulmonary embolism.

The mean cost for a Quixil dose is comparable to the use of one Di-deco device for each patient; in addition, one operator is necessary to wash and prepare blood units from the Di-deco.

We therefore believe that use of fibrin tissue adhesive is advantageous, as it reduces blood loss; avoiding bleeding has been shown to be safer than reinfusion, since even autologous blood is at risk of contamination. Furthermore, one operator is needed to prepare units, increasing theoretical costs.

In conclusion, use of fibrin tissue adhesive was found to significantly reduce apparent blood loss, also decreasing costs. On comparison of fibrin glue use with postoperative blood reinfusion from the Di-deco device in TKR, we noted a decrease of apparent blood loss, a similar decrease of hemoglobin values, and transfusions of fewer blood units, even if it is not possible to define a real decrease of transfused units due to the variability of patient features.

We believe that fibrin tissue adhesive can be an appropriate solution to enhance hemostasis and vessel sealing at the operative site in order to reduce blood loss and patient morbidity after surgery.
